# Limonene in exhaled breath is elevated in hepatic encephalopathy

**DOI:** 10.1088/1752-7155/10/4/046010

**Published:** 2016-11-21

**Authors:** M E O’Hara, R Fernández del Río, A Holt, P Pemberton, T Shah, T Whitehouse, C A Mayhew

**Affiliations:** 1 School of Physics and Astronomy, University of Birmingham, Birmingham B15 2TT, UK; 2 Department of Hepatology, University Hospital Birmingham NHS Trust, Birmingham B15 2TH, UK; 3 Critical Care and Anaesthesia, University Hospital Birmingham NHS Trust, Birmingham B15 2TH, UK; 4 Institut für Atemgasanalytik, Leopold-Franzens-Universitaet Innsbruck, Rathausplatz 4, 6850 Dornbirn, Austria; 5 Author to whom any correspondence should be addressed.; m.e.ohara@bham.ac.uk; r.fernandezdelrio@bham.ac.uk; c.mayhew@bham.ac.uk; Andrew.holt@uhb.nhs.uk; Tahir.Shah@bham.ac.uk; pembo@doctors.org; Tony.Whitehouse@uhb.nhs.uk

**Keywords:** breath analysis, cirrhosis, limonene, hepatic encephalopathy, PTR-MS, volatile organic compound

## Abstract

Breath samples were taken from 31 patients with liver disease and 30 controls in a clinical setting and proton transfer reaction quadrupole mass spectrometry (PTR-Quad-MS) used to measure the concentration of volatile organic compounds (VOCs). All patients had cirrhosis of various etiologies, with some also suffering from hepatocellular cancer (HCC) and/or hepatic encephalopathy (HE). Breath limonene was higher in patients with No-HCC than with HCC, median (lower/upper quartile) 14.2 (7.2/60.1) versus 3.6 (2.0/13.7) and 1.5 (1.1/2.3) nmol mol^−1^ in controls. This may reflect disease severity, as those with No-HCC had significantly higher UKELD (United Kingdom model for End stage Liver Disease) scores. Patients with HE were categorized as having HE symptoms presently, having a history but no current symptoms and having neither history nor current symptoms. Breath limonene in these groups was median (lower/upper quartile) 46.0 (14.0/103), 4.2 (2.6/6.4) and 7.2 (2.0/19.1) nmol mol^−1^, respectively. The higher concentration of limonene in those with current symptoms of HE than with a history but no current symptoms cannot be explained by disease severity as their UKELD scores were not significantly different. Longitudinal data from two patients admitted to hospital with HE show a large intra-subject variation in breath limonene, median (range) 18 (10–44) and 42 (32–58) nmol mol^−1^.

**Abbreviations**
ALD: Alcoholic liver disease
GC: Gas chromatography
HE: Hepatic encephalopathy
HCC: Hepatocellular cancer
ITU: Intensive treatment unit
LQ: Lower quartile
MS: Mass spectrometry
MHE: Minimal hepatic encephalopathy
OHE: Overt hepatic encephalopathy
OPU: Out-patient clinic
ppbv: Parts per billion by volume
PTR-MS: Proton transfer reaction mass spectrometry
UKELD: United Kingdom model for End-stage Liver Disease
UQ: Upper quartile
VOC: Volatile organic compounds
VMR: Volume mixing ratio

## 1. Introduction

Volatile organic compounds (VOCs) are produced in the body as a result of various metabolic processes and endogenous VOC analysis in the breath has been proposed as a potential diagnostic tool in human disease. Breath analysis has attracted clinical and scientific attention as a potential means for delivering non-invasive, real-time rapid screening and diagnosis of complex diseases. Recent overviews of the field are provided in a book published in 2014 [1] and in numerous review papers [2–6].

Breath VOCs have been measured in patients with liver disease in a number of studies [7–21] and several VOCs have been suggested as biomarkers for cirrhosis. In particular, sulphur compounds have been suggested as being associated with liver disease and are responsible for the characteristic odour of patient’s breath called Foetor Hepaticus [19, 22]. Previous work has included patients with various etiologies, different sampling methods and different analytical methods. It is, therefore, difficult to compare results. There is some agreement as to which potential biomarkers are associated with liver disease, but not all studies find the same volatile markers. As previously reported [7], these issues were addressed by conducting a two phase biomarker discovery study. Limonene, methanol and 2-pentanone were identified as biomarkers of liver cirrhosis. These VOCs not only discriminated cases from controls but were also significantly different before and after liver transplantation in a sub-group of patients who had received a transplant.

Pilot data [23] was obtained by our group prior to the published study from patients in out-patient clinic. Twelve had cirrhosis or fibrosis, four had neuroendocrine disease with liver tumours and five had well-functioning graft livers following previous liver transplants due to Hepatitis C virus. This study found that a monoterpene, which was assumed to be limonene, had elevated concentrations in the breath of patients with cirrhosis, and in particular, those with hepatic encephalopathy (HE). Limonene in the breath of patients with liver cirrhosis has been observed in two other studies using GC-MS [9, 10] and tentatively identified in a proton transfer reaction—time of flight-mass spectrometric (PTR-TOF-MS) study [13]. Limonene is a monoterpene of dietary origin, typically found in citrus fruits but also naturally occurring in many vegetables. There is no evidence to suggest that it can be produced in the human body. It is used in the food and drink industry to give a citrus flavor and in perfumes and air fresheners. As such, it is a ubiquitous exogenous compound and it would be difficult to avoid in the diet in general. Limonene is metabolized in the liver by the P450 enzymes CYP2C9 and CYP2C19 [24] to the metabolites perillyl alcohol, trans-carveol and trans-isopiperitenol. It has been noted that levels of the enzyme CYP2C19 are reduced in patients with cirrhosis and, that levels inversely correlate with severity of cirrhosis [25]. This presents the possibility that elevated levels of limonene in the body may be associated with liver disease owing to a reduced ability of the liver to produce the appropriate metabolic enzymes, as has already been noted [13].

The present work will discuss these results further in respect to HE, a co-morbidity of advanced liver cirrhosis. Comparisons of cirrhosis with and without hepatocellular cancer (HCC) and HE will be shown. Results of longitudinal data on patients with HE to examine intra-individual variation in these three VOCs of interest will also be shown.

HE is a spectrum of neuropsychiatric abnormality associated with liver dysfunction. Symptoms vary in severity depending on the stage of the illness and include confusion, lethargy, sleep disturbance, personality change and cognitive impairment. In its extreme form, it can lead to coma and even death [26]. Overt HE (OHE) can be diagnosed clinically, usually using a scoring system such as the West-Haven criteria, and can be episodic or persistent. HE can affect the patient’s mental functioning intermittently [27], so it is possible for them to pass a neurological test on the day of the examination, while still suffering from symptoms at other times.

Minimal HE (MHE) is difficult to diagnose and requires specialized testing. It is possible for patients to be suffering from undiagnosed MHE [28]. There is no gold standard for the diagnosis of HE and many scoring systems have been criticized for lack of objectivity [28]. Current recommendations [29] include using the clinical HE staging scale (CHESS) [30], the HE scoring algorightm (HESA) [31] or modified orientation log (MOD) to refine the West-Haven criteria but their widespread use has yet to be adopted.

Estimates of the incidence of MHE range from 20% to 84% of cirrhotic patients, depending on which testing methods are used [27]. HE not only degrades quality of life, but it means a poorer prognosis for the patient [32]. HE is thought to be caused by the action of neurotoxic substances including ammonia and manganese, which are usually metabolised by the liver, persisting in elevated concentrations. These toxins can then cause glutamine induced changes in astrocytes that lead to the clinical manifestations of HE [33, 34]. Usual treatment is through the use of laxatives and antibiotics which act to prevent the accumulation of toxins. In the advanced stages, patients must be admitted to hospital for management of episodes of OHE.

Breath analysis for HE has been investigated previously in two other pilot studies which used GC-MS in patients with cirrhosis [20, 35], but neither of these reported limonene as a marker for HE. One pilot study investigated HE using an e-nose [36], but this technique is unable to specify exactly which VOCs are responsible for the discrimination so it is not possible to determine whether it is based on limonene.

## 2. Methods

### 2.1. Patients, controls and hospital room air

Patients were recruited at the University Hospital Birmingham from either the transplant assessment clinic or in wards after being admitted with HE. The regional ethics committee of Camden and Islington, London approved this study (REC reference: 13/LO/0952). Written informed consent was obtained from each volunteer.

Thirty-one patients suffering from liver disease participated in the pre-transplant measurements (F/M 8/23, mean age 55 years, min–max 27–71 years). There were a number of etiologies and 11 patients had more than one condition. Full details of the patients and their diagnoses are given in our earlier publication which reported volatiles associated with liver cirrhosis.

For the longitudinal data, two patients were recruited having been admitted as in-patients suffering from HE. Patient M1 was a 54 years old male with a primary diagnosis of alcoholic liver disease (ALD). He gave four breath samples during an admission for HE before his liver transplant, then three further samples after transplant. Patient M15 was also a 54 years old male with a primary diagnosis of ALD. He gave breath samples on seven different days during an admission for HE. He did not receive a transplant so gave no post-transplant samples.

For 28 pre-transplant measurements, breath samples from the patients’ companions were taken. For the other three, two came alone to clinic and the other’s companion declined to take part. Two additional controls were therefore recruited, one was a ward nurse and the other was a visitor to the hospital in ITU. These controls, while not related to the patient, had been in the same room for several hours prior to sampling so that confounding factors associated with any volatiles present in the room environment could be taken into consideration. In total 30 controls (F:M 23:7, mean age 44 years, min–max 20–75 years) took part in the study. The larger number of females in the control group arose because the majority of patients were men and often attended the clinic with their wives. While this means the control group is not ideally matched, there is no consistent evidence of dependences of volatile breath composition on sex [1, 37]. In confirmation of this, no correlation was found between sex and VOCs in either our control or patient groups. It was considered that inhaled VOCs have a greater potential to confound biomarker discovery. As the majority of the companions were living with the patients, they provided an ideal control for exposure of exogenous volatiles in the home environment. VOCs inhaled at home, or in transit, may well still be present in breath for hours or days after inhalation; the biological half-life of inhaled VOCs are not well known and in any case may be patient-specific [38].

All study subjects were asked to complete a detailed questionnaire which included details on their home environment, diet, smoking status, health and medications. Participants were asked if they had consumed fruit, fruit juices and fruit-flavoured drinks as a normal part of their diet, and, if so, to provide details on quantity and how long before the breath sampling these had been consumed.

Hospital room air was collected every time breath samples were taken so that any exogenous volatiles, such as isopropanol coming from hand gels, resulting in product ions at *m*/*z* 43 and *m*/*z* 61, could be taken into consideration. There are fewer room air samples than patient-control pairs because the same room was used for consecutive patient-control pairs on the same day in some cases.

Diagnosis of HCC was based on previous investigations including radiology and histology. For analysis of HCC, the groupings are **HCC** (*N*  =  10): those with at least one hepatocellular tumour, and **No-HCC** (*N*  =  21): those with no hepatocellular tumours. HE status was assessed both from clinical diagnosis using the West Haven criteria and also from questioning of the patient and the relative with whom they were attending clinic. Patients and relatives were questioned as to whether the patient was suffering from any symptoms of HE at the present time and were classed as having HE at the present time if they answered yes. Some patients and relatives reported having had HE in the past, sometimes following a trigger such as an infection, but that the patient had recovered and was not symptomatic on the day of the breath sample. These patients were classed as having a history but not currently suffering. The final group of patients had never had HE and did not have symptoms on the day of sampling according to their medical records and their own and their companions’ assessment of their mental function. These patients are classed as ‘No-HE’. For analysis of HE, the grouping are: 1. **HE-now:** suffering symptoms of HE on the day the breath samples were taken (*N*  =  11); 2. **History-HE:** had suffered from HE in the past but were not suffering symptoms on the day of sampling (*N*  =  7), and 3. **No-HE**: those who were not suffering symptoms on the day of sampling, nor had any history of HE (*N*  =  11). All patients were ambulatory and were seen in clinic, apart from four who had been admitted to hospital for HE, one of whom had acute liver failure. Only one patient (ID code M15) had HE above grade 2. In the No-HE and History-HE groups, no patients were taking medication for HE. One patient in each of these two groups was taking lactulose, but in each case this was for constipation, not for symptoms of HE. Of the 11 HE-now patients, eight were taking medication for HE: three were on lactulose and rifaximin, one on rifaximin only and four on lactulose only.

### 2.2. Breath sampling protocol

**Figure 1.**
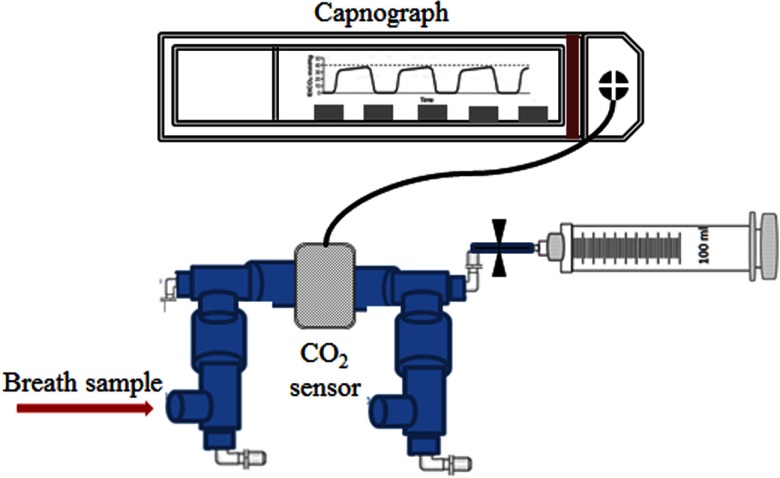
Schematic of the breath sampling device. Breath samples are only drawn into the glass syringe once the capnograph shows that the alveolar phase of the exhaled breath has been reached. Typically 3–4 breaths are needed to fill a 100 ml syringe.

There is no agreed standard for the collection of breath for volatile analysis, and uncontrolled breath sampling has been shown to be unreliable [39, 40]. Therefore, capnography controlled sampling was used to collect only the alveolar phase of the breath. Subjects were in a relaxed state throughout the measurements and were either in a seated or recumbent position. They were asked to breathe normally into a gas tight respiratory system (Intersurgical Limited) containing an in-line CO_2_ mainstream sensor connected to a fast-time response capnometer (Capnogard 1265 Novametrix Medical Systems Inc.). A 100 ml glass syringe (Sigma-Aldrich) was coupled to the tubing using a 3-way luer-lock stopcock (Braun Medical Limited). When the alveolar plateau on the capnograph was observed, a breath sample was manually drawn from the subject’s breath stream into the syringe. Three to four alveolar breath samples were collected for each 100 ml syringe, and four replicates of these were taken for each subject. Glass syringes were used, because our tests showed that they have no contaminating volatiles. Figure 1 schematically shows the sampling system used.

After collection, the syringes were sealed using the luer lock fitting. They were transported from hospital to laboratory (a 10 min outdoor walk) in an opaque storage box. Once at the laboratory, the syringes were placed inside an incubator set at 40 °C.

All samples were mass spectrometrically analyzed within 2 h of collection. For the measurements, syringes were taken out of the incubator and immediately placed into a purpose designed heating bag (Infroheat, Wolverhampton) maintained at a constant temperature of 40 °C in order to limit condensation, which could otherwise lead to volatile loss [40]. The luer stopcock was coupled to a Swagelok fitting and connected directly to the inlet of the analytical device, a proton transfer reaction mass spectrometer (PTR-MS). The inlet flow was set at 10–15 ml min^−1^ and the drift tube and inlet lines were maintained at 45 °C. The syringes are gas tight and have minimal friction such that atmospheric pressure is sufficient to push the plunger in smoothly so that the breath sample is being drawn into the instrument at a constant flow.

### 2.3. Analytical measurements

PTR-MS is a platform technology designed to detect low concentrations of volatiles (less than parts per billion by volume). Hence it has found use in many analytical applications ranging from drug detection through to industrial pollution [1, 41–44]. Details of the instrument used, a PTR-Quad-MS (IONICON Analytik GmbH), and how it operates are described in detail in the literature [1, 40, 41]. In brief, it exploits the reactions of protonated water with neutral volatiles (M), usually leading to a protonated parent (MH^+^). If dissociative proton transfer occurs then it is not extensive in terms of the number of resulting product ions. Operational parameters used for this investigation were those previously reported [45, 46]. Namely, the drift-tube was maintained at a pressure of 2.07  ±  0.01 mbar and temperature of 45  ±  1 °C. The voltage across the drift-tube was set at 600 V, which is sufficiently high to reduce water clustering to reagent and product ions by collision induced dissociation.

A *m*/*z* range of 20–200 amu was scanned with a dwell time of 0.5 s per atomic mass unit. Mass spectra of the breath samples were recorded from the average of three cycles for each of the four syringes, for every participant. These four spectra were averaged to provide one data set for each subject with the uncertainty expressed as the standard error of the mean for the four syringes.

The intensities of the product ion(s) associated with a given volatile were converted from normalized counts per second (cps) to volume mixing ratios (VMR) in units of nmol mol^−1^. Normalisation was to 50 million cps of the sum of the area under the peak of the H_3_O^+^ and the (H_3_O^+^) · (H_2_O) ions. Conversion to VMR was by use of a standard procedure that relies on a calculated, compound-specific, collisional reaction rate coefficient, determined using the effective translational temperature of the reagent ions [1]. It should be noted that PTR-Quad-MS has a mass resolution of approximately 1 amu so it is possible that other compounds or fragments could contribute to observed peaks.

To help identify product ions, pure samples of key volatiles were individually measured using PTR-MS to establish the *m*/*z* values of the product ions.

#### 2.3.1. Statistical analyses

The data sets for each volatile of interest were assessed using a Shapiro–Wilks test and were found not to be normally distributed so non-parametric tests were used. IBM SPSS version 22 was used for all statistical analysis. Mann–Whitney U-tests determined which *m*/*z* values differed between the patients and controls in the original study, and to assess whether concentrations of volatiles were significantly different between HCC with non-HCC patients. A Kruskal–Wallis one way analysis of variance was used to compare the HE-now, History-HE and No-HE groups. All tests were done using a significance level of 0.95.

## 3. Results and discussion

### 3.1. Case control study

As previously reported [7], differences between patients and controls were found for four mass spectral peaks: *m*/*z* 33, *m*/*z* 81, *m*/*z* 87 and *m*/*z* 137. Peaks at *m*/*z* 81 and *m*/*z* 137 result from limonene; the protonated parent is at 137 and *m*/*z* 81 is a fragment ion (C_6_H_9_) [47, 48]. The area under the peaks for both *m*/*z* 81 and *m*/*z* 137 were summed and used to determine the concentration of limonene in the breath samples. PTR-MS is unable to differentiate isomers, and there is a possibility that other monoterpenes could have contributed to these peaks. However, previous work in humans has indicated that limonene is the most abundant monoterpene in human breath and blood [49]. GC-MS confirmation was performed with one of the breath samples to confirm the presence of limonene by colleagues at the Max Planck Institute in Mainz, Germany. The methodology has been previously reported [50, 51]. Limonene has also been shown to be elevated in the breath of patients with liver disease in other studies [10, 13].

**Figure 2.**
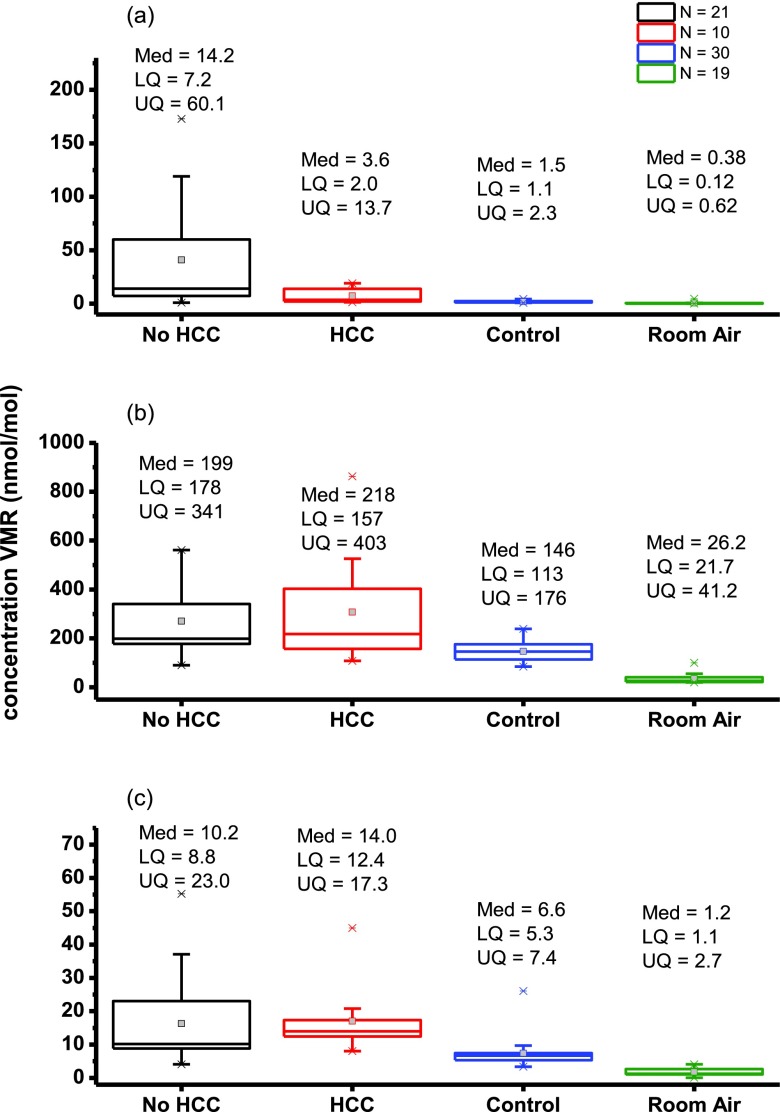
Boxplots showing in units of nmol mol^−1^ lower quartile (LQ), median (Med), and upper quartile (UQ) calculated volume mixing ratios (VMRs) for (a) limonene, (b) methanol and (c) 2-pentanone for 21 patients with liver cirrhosis without HCC (No-HCC), 10 patients with cirrhosis and HCC (HCC), 30 controls and room air. Whiskers are 1.5 times the inter-quartile range and outliers are depicted by a star.

The patient group has been further subdivided into those with and without hepatocellular cancer (HCC and No-HCC, respectively. Box plots of limonene, methanol (*m*/*z* 33) and 2-pentanone (*m*/*z* 87) for patients with and without HCC, controls and room air are shown in figure 2. Figures for mean, (lower quartile (LQ)/upper quartile (UQ)) are given in the figure. There is a difference between patients with and without HCC only for limonene, with a Mann–Whitney U score of significance *p*  =  0.015.

**Figure 3.**
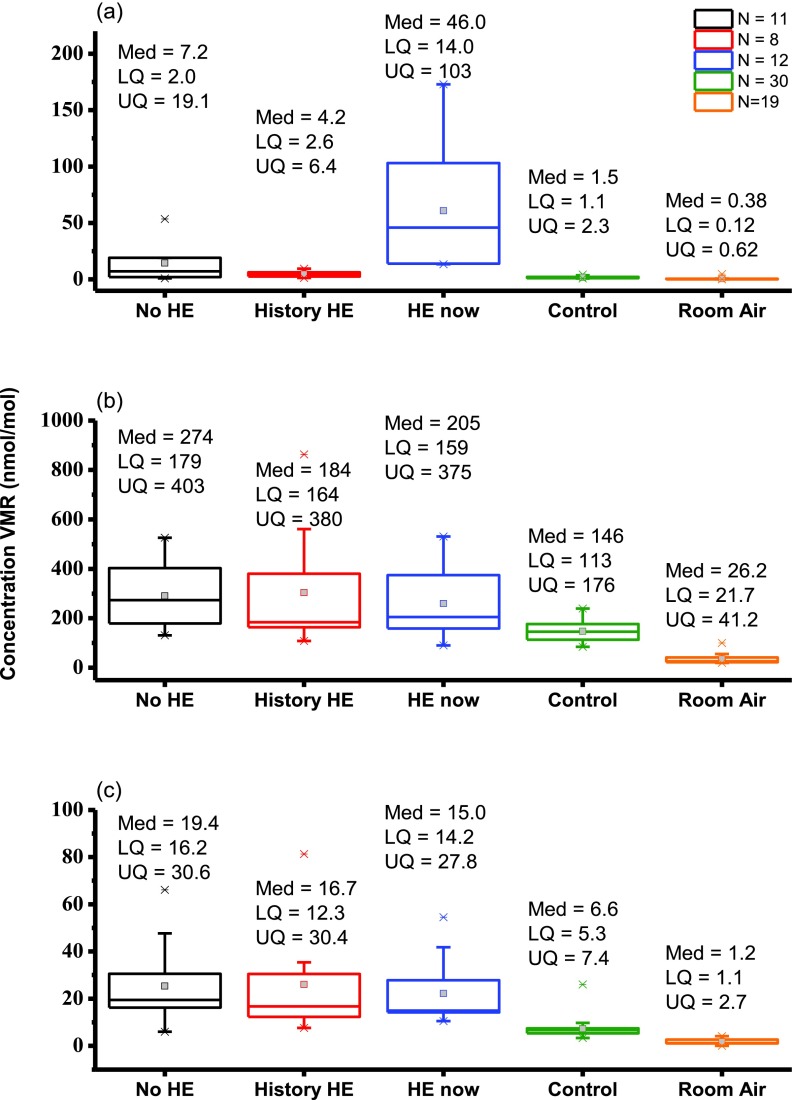
Boxplots showing in units of nmol mol^−1^ lower quartile (LQ), median (Med) and upper quartile (UQ) calculated volume mixing ratios (VMRs) for (a) limonene, (b) methanol and (c) 2-pentanone for 11 patients without HE (No HE), 8 patients who have a history of HE but are not showing symptoms on the day of sampling (History HE), 12 patients who were suffering symptoms of HE on the day of sampling (HE-now), 30 controls and room air. Whiskers are 1.5 times the inter-quartile range and outliers are depicted by a star.

Figure 3 shows comparisons for the groups No-HE, History-HE, HE-now, controls and room air. Comparison of the three patient groups revealed that only one VOC, limonene, was able to successfully differentiate the groups with a Kruskall–Wallis score of significance *p*  =  0.001. Figure 3 shows a clear difference between HE-now and History-HE. For No-HE, there is overlap with the HE-now group, but this is highly skewed by just three patients.

It was conjectured that the difference in means between breath limonene in the No-HCC versus HCC groups may be a reflection of severity of cirrhosis. Often patients are considered for transplant on the basis of the presence of a hepatocellular tumour while the rest of the liver is still compensating adequately. This conjecture is supported by analysis of the UKELD (United Kingdom model for End-stage Liver Disease) score for the groups. UKELD is a scoring algorithm which combines a selection of clinical chemistry values and gives a prediction of 1 year mortality [52]. It is used to select patients for transplant in the United Kingdom and patients are usually considered for transplant when their UKELD score is  >49, unless there is an additional indication for transplant such as HCC. The mean (range) of the UKELD score for the No-HCC and HCC groups is 54 (48–63) and 49 (43–56), respectively. These are significantly different (Mann–Whitney *p*  =  0.002). A similar analysis was done for the HE classifications to determine whether patients in the HE-now group had more severe disease. UKELD scores for the three groups were 49 (43–57) for No-HE, 53 (46–63) for History-HE and 55 (51–62) for HE-now. There is no significant difference between the UKELD scores for the History-HE and HE-now groups (Mann–Whitney *p*  =  0.21), but there is a significant difference between the No-HE groups and the HE-now groups (Mann–Whitney *p*  =  0.001). It is perhaps not surprising that the group which has never experienced HE has a lower UKELD score than the group which was suffering symptoms at the time of sampling. However, the large difference in limonene between the History-HE group and the HE-now group cannot be explained by a difference in severity as judged by UKELD score. Overall, as previously reported [7], there is no correlation between the concentration of breath limonene and UKELD score. A prospective observational multicentre study of 1348 patients in 29 liver units in eight European countries was recently undertaken to investigate risk factors for the development of HE and assess survival in patients with liver cirrhosis [53]. This found that previous HE was the most important risk factor for the development of HE, with a poor relationship between traditional precipitating factors such as bacterial infections, active alcoholism or gastrointestinal haemorrhage. Our present study has found that limonene is able to distinguish between History-HE and HE-now groups, which presents the possibility of a screening or monitoring tool to enable targeting of prophylactic therapies in at-risk populations.

As previously reported [7], there was no association between self-reported consumption of fruit/fruit juice and elevated breath limonene. Studies by us (unpublished) have shown that in normal healthy people, consumption of pure, concentrated orange juice is followed by a small increase in breath limonene which returns to baseline within half an hour. There is nothing in the diet or previous 24 h diet of the patients which would explain elevated breath limonene.

Khalid *et al* suggested isothiocyanato-cyclohexane as indicating the presence of HE, and methyl vinyl ketone as indicating absence of HE within a group of alcoholic cirrhotics [35]. They used a presence/absence analysis in which absolute concentrations were not measured; rather only the presence or absence of a compound above three times the signal to noise ratio was compared across groups. Using this analysis, our previous results would not have shown a difference between cases and controls because limonene was also observed in controls, albeit at very low concentrations. Limonene has also been found in the blood and in the emissions from skin of healthy volunteers, and can be considered a normal volatile emission from healthy humans [54, 55]. A previous study using GC-MS to examine the normal volatile constituents of human breath reported limonene in the venous blood and breath of all 28 subjects [49]. The mean (range) concentration in breath was 1.46 ppbv (0.27–7.42), which is similar to our findings.

**Figure 4.**
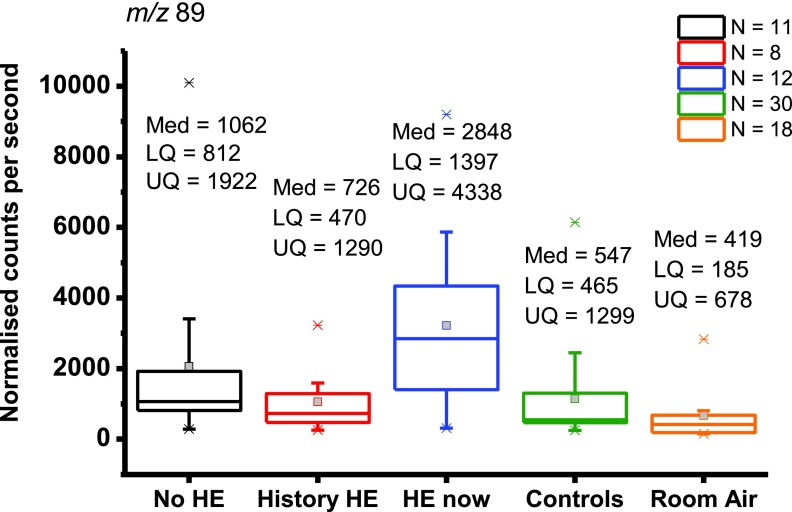
Boxplots showing normalized counts per second lower quartile (LQ), median (Med) and upper quartile (UQ) calculated volume mixing ratios (VMRs) for *m*/*z* 89 for 11 patients without HE (No-HE), 8 patients who have a history of HE but are not showing symptoms on the day of sampling (history HCC), 12 patients who were suffering symptoms of HE on the day of sampling (HE-now), 30 controls and room air. Whiskers are 1.5 times the inter-quartile range and outliers are depicted by a star. Note that only 18 room air samples are used as one sample gave a peak which could not be distinguished above the instrumental noise.

To our knowledge, Vollnberg *et al* [20] remains, until now, a conference abstract rather than a full paper so insufficient details are known to make a comparison, but they noted acetoin (3-hydroxybutanone) and 2-pentanone as having predictive utility for HE [20]. The present study has found 2-pentanone as predictive for the presence of cirrhosis versus controls, but did not find a difference between those with and without HE. Participants had been asked to indicate when they last had something to eat or drink, but there was no association with time since last meal and concentration of 2-pentanone. Acetoin would give a protonated parent peak at *m*/*z* 89. In our initial case-control comparison, *m*/*z* 89 was significantly different between patients and controls. It did not, however, show a difference between patients pre- and post-transplant so it was discounted as being a useful diagnostic volatile for liver cirrhosis. Figure 4 shows the box plot for groupings of HE status for *m*/*z* 89. Only normalized counts per second are shown as we are not confident about assigning an identity to this peak. The HE-now group is significantly different from the other groups, but the variation in room air is large and it overlaps with all other groups. An examination of the 12 patients who had a transplant shows that the concentration of *m*/*z* 89 in patients with and without HE is not significantly different. It is important to bear in mind that isomeric and isobaric compounds cannot be distinguished in our system and a signal from acetoin may be confounded by the presence of another volatile which produced a product ion at the same *m*/*z*.

### 3.2. Longitudinal study

**Figure 5.**
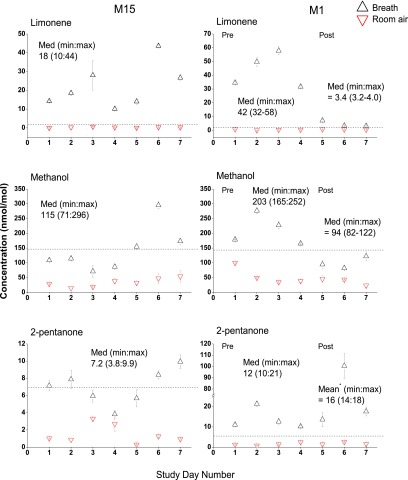
Scatter plots showing in units of nmol mol^−1^ lower quartile (LQ), median (Med) and upper quartile (UQ) calculated volume mixing ratios (VMRs) for (a) limonene, (b) methanol and (c) 2-pentanone for patients M15 (left hand panel) and M1 (right hand panel). Breath concentrations are shown in black triangles and room air in red triangles. For M1, study days 1–4 are pre-transplant and days 5–7 are post-transplant. For patient M15, all days are pre-transplant as he did not receive a transplant. The dashed horizontal line indicates the median of the concentration for healthy controls for each particular volatile. ^*^The mean is given for M1 2-pentanone as it does not include the outlying point.

Two patients (ID codes M1 and M15) who had been admitted suffering with HE were followed over the course of their stay in hospital. Only M15 had high grade HE, and had very low mental capacity. M1 received a liver transplant during the course of the study so his results show four pre-transplant samples and three post-transplant. Figure 5 shows concentrations of limonene, methanol and 2-pentanone in breath and room air over the course of the measurement period. For M1, study days 1, 2, 3 and 4 correspond to 47, 44, 42 and 40 d prior to transplant, respectively. Study days 5, 6 and 7 correspond to 28, 29 and 33 d after transplant. All measurements were taken in wards, except for study day 7 which was taken in the out-patient clinic. For M15, if study day 1 is taken to be on day 1, study days 1–7 correspond to days 1, 2, 6, 7, 8, 9 and 15. The patient was discharged on day 15.

The main thing to note is the high degree of variability from day to day. Despite this, there is still a measureable drop in breath limonene and methanol following transplant for M1. There is what appears to be an anomalous result for 2-pentanone for M1 on study day 6, which is, at present, unexplained as it was also not present in the room air. A corresponding peak in the mass spectrum at *m*/*z* 89 suggests that it may have been a chlorinated compound. It is possible that the patient inhaled an environmental contaminant at high concentration some time before the breath test and it was still washing out.

The variability for M15 is more pronounced than for M1. This patient had grade 3 or 4 HE for the first 6 d of the study. On study day 7 his acute HE had resolved sufficiently to be discharged later that day, although it is likely that he was still suffering from minimal HE as he had a recent history of repeated hospital admissions. There were difficulties in obtaining samples from this patient on some days of the study as his mental incapacity was such that he was unable at times to comply with instructions to breathe into the apparatus. It was, therefore, very difficult to obtain reliable end-tidal samples and it is possible that some of his samples were contaminated with dead-space air. From this limited evidence, it does not appear that breath limonene can predict daily variations in symptoms of HE for an individual patient.

## 4. Conclusions

Limonene, methanol and 2-pentanone have previously been shown to be elevated in the breath of cirrhotic patients compared with controls and in patients pre-transplant compared with the same patients after transplant [7]. Limonene, but not methanol or 2-pentanone is able to discriminate patients currently suffering symptoms of HE from patients with no current symptoms. Patients with HCC had lower levels of limonene than those without HCC but this may be a reflection of the fact that patients with HCC are usually considered for transplant at an earlier stage of liver decompensation. This is supported by the fact that the UKELD scores for patients with HCC were significantly lower than patients without HCC.

It is possible that breath limonene is higher in more severe liver disease, and because HE is a complication of advanced disease, high breath limonene may simply be a proxy for advanced disease. However, there was no correlation between limonene and any clinical chemistry value. Nor was there a scorrelation with UKELD score. This leaves the possibility that limonene is itself a causative agent in HE. Limonene is highly lipophilic; this, together with its low molecular mass, make it possible for it to cross the blood–brain barrier. There have been cases of acute poisonings with tea tree oil, which is composed of terpenes and terpinoids, including limonene at low levels. The symptoms were confusion, drowsiness and coma [56]. These are strikingly similar to the symptoms of HE, and suggest that molecules from the oil crossed the blood–brain barrier to cause neurological symptoms. In all cases, the effects were transient and the patients recovered within a matter of hours. While this was not pure limonene, terpinoid oils are composed of similar units and it is not known what biochemical reactions will occur if limonene is resident in the human body for long periods. While limonene has not been shown to be acutely toxic in humans [57], information is lacking about chronic exposure. If a cirrhosis patient lives with a high blood concentration of limonene for months or even years, it is not known how this will affect their health.

It has been shown that limonene concentrates in human breast tissue in women given a large daily dose of limonene for 2 weeks before undergoing surgery for breast cancer [58]. Breast tissue is composed mainly of fat [59] so this lends weight to our previously stated hypothesis [7] that limonene, being highly lipophilic, accumulates in the fat tissue of patients with liver disease. The wash-out curves for patients who gave breath samples on consecutive days in the 3 weeks after liver transplant presented in our previous paper [7] support this hypothesis. The brain has a high fat content, so it would be expected that limonene would partition into it should it cross the blood–brain barrier.

The main limitation of this study is the small sample size. The practical difficulty of requiring a patient with severe HE to comply with instructions would have to be addressed in future studies. The evidence presented here suggests that breath limonene is an exogenous compound which may serve as an indicator of an elevated risk of HE in patients with cirrhosis of the liver. The hypothesis that limonene is itself in the causative pathway of HE remains to be explored.
